# Supplementary material for: Sequence and immunologic conservation of Anaplasma marginale OmpA within strains from Ghana as compared to the predominant OmpA variant
Source: PLoS One. 2019 Jul 10;14(7):e0217661. doi: 10.1371/journal.pone.0217661 (PMC6619652; doi:10.1371/journal.pone.0217661)
Supplement: S1 Fig — Ghanaian OmpA variants include GV1, GV2 and GV3. OmpA_V1 represents St. Maries, Virginia, Kansas 6DE, Colville C51 and C52, and Nayarit-Mexico N3574. OmpA_V2 represents Puerto Rico, Dawn-Australia, and Nayarit-Mexico N4506. OmpA_V3 represents Kansas EMΦ. (RTF) [file pone.0217661.s001.rtf]

OmpA_V1     1 ATGCTGCATCGTTGGTTAGCTCTGTGCTTCTTGGCATCGTTTGCCGTGACAGGTTGTGGT
OmpA_V2     1 ATGCTGCATCGTTGGTTAGCTCTGTGCTTCTTGGCATCGTTTGCCGTGACAGGTTGTGGT
OmpA_GV3    1 ATGCTGCATCGTTGGTTAGCTCTGTGCTTCTTGGCATCGTTTGCCGTGACAGGTTGTGGT
OmpA_V3     1 ATGCTGCATCGTTGGTTAGCTCTGTGCTTCTTGGCATCGTTTGCCGTGACAGGTTGTGGT
OmpA_GV1    1 ATGCTGCATCGTTGGTTAGCTCTGTGCTTCTTGGCATCGTTTGCCGTGACAGGTTGTGGT
OmpA_GV2    1 ATGCTGCATCGTTGGTTAGCTCTGTGCTTCTTGGCATCGTTTGCCGTGACAGGTTGTGGT


OmpA_V1    61 CTTTTCAGCAAGGAAAAGGTCGGGATGGACATTGTCGGCGTGCCTTTTAGCGCTGGGAGG
OmpA_V2    61 CTTTTCAGCAAGGAAAAGGTCGGGATGGACATTGTCGGCGTGCCTTTTAGCGCTGGGAGG
OmpA_GV3   61 CTTTTCAGCAAGGAAAAGGTCGGGATGGACATTGTCGGCGTGCCTTTTAGCGCTGGGAGG
OmpA_V3    61 CTTTTCAGCAAGGAAAAGGTCGGGATGGACATTGTCGGCGTGCCTTTTAGCGCTGGGAGG
OmpA_GV1   61 CTTTTCAGCAAGGAAAAGGTCGGGATGGACATTGTCGGCGTGCCTTTTAGCGCTGGGAGG
OmpA_GV2   61 CTTTTCAGCAAGGAAAAGGTCGGGATGGACATTGTCGGCGTGCCTTTTAGCGCTGGGAGG


OmpA_V1   121 GTAGAAAAGGTTTATTTTGACTTCAACAAGTACGAAATAAAGGGTTCTGGCAAGAAGGTG
OmpA_V2   121 GTAGAAAAGGTTTATTTTGACTTCAACAAGTACGAAATAAAGGGTTCTGGCAAGAAGGTG
OmpA_GV3  121 GTAGAAAAGGTTTATTTTGACTTCAACAAGTACGAAATAAAGGGTTCTGGCAAGAAGGTG
OmpA_V3   121 GTAGAAAAGGTTTATTTTGACTTCAACAAGTACGAAATAAAGGGTTCTGGCAAGAAGGTG
OmpA_GV1  121 GTAGAAAAGGTTTATTTTGACTTCAACAAGTACGAAATAAAGGGTTCTGGCAAGAAGGTG
OmpA_GV2  121 GTAGAAAAGGTTTATTTTGACTTCAACAAGTACGAAATAAAGGGTTCTGGCAAGAAGGTG


OmpA_V1   181 CTGCTGGGCCTGGTAGAAAGAATGAAGGCGGACAAGAGGTCCACCCTGCTGATTATCGGC
OmpA_V2   181 CTGCTGGGCCTGGTAGAAAGAATGAAGGCGGACAAGAGGTCCACCCTGCTGATTATCGGC
OmpA_GV3  181 CTGCTGGGCCTGGTAGAAAGAATGAAGGCGGACAAGAGGTCCACCCTGCTGATTATCGGC
OmpA_V3   181 CTGCTGGGCCTGGTAGAAAGAATGAAGGCGGACAAGAGGTCCACCCTGCTGATTATCGGC
OmpA_GV1  181 CTGCTGGGCCTGGTAGAAAGAATGAAGGCGGACAAGAGGTCCACCCTGCTGATTATCGGC
OmpA_GV2  181 CTGCTGGGCCTGGTAGAAAGAATGAAGGCGGACAAGAGGTCCACCCTGCTGATTATCGGC


OmpA_V1   241 CATACAGACTCCAGGGGAACGGAAGAGTACAACCTGGCGCTGGGTGAGAGAAGGGCAAAT
OmpA_V2   241 CATACAGACTCCAGGGGAACGGAAGAGTACAACCTGGCGCTGGGTGAGAGAAGGGCAAAT
OmpA_GV3  241 CATACAGACTCCAGGGGAACGGAAGAGTACAACCTGGCGCTGGGTGAGAGAAGGGCAAAT
OmpA_V3   241 CATACAGACTCCAGGGGAACGGAAGAGTACAACCTGGCGCTGGGTGAGAGAAGGGCAAAT
OmpA_GV1  241 CATACAGACTCCAGGGGAACGGAAGAGTACAACCTGGCGCTGGGTGAGAGAAGGGCAAAT
OmpA_GV2  241 CATACAGACTCCAGGGGAACGGAAGAGTACAACCTGGCGCTGGGTGAGAGAAGGGCAAAT


OmpA_V1   301 GCTGTTAAGGAGTTCATACTAGGGTGCGACAGGTCGCTATCCCCTAGAATATCTACTCAA
OmpA_V2   301 GCTGTTAAGGAGTTCATACTAGGGTGCGACAGGTCGCTATCCCCTAGAATATCTACTCAA
OmpA_GV3  301 GCTGTTAAGGAGTTCATACTAGGGTGCGACAGGTTGCTATCCCCTAGAATATCTACTCAA
OmpA_V3   301 GCTGTTAAGGAGTTCATACTAGGGTGCGACAGGTCGCTATCCCCTAGAATATCTACTCAA
OmpA_GV1  301 GCTGTTAAGGAGTTCATACTAGGGTGCGACAGGTCGCTATCCCCTAGAATATCTACTCAA
OmpA_GV2  301 GCTGTTAAGGAGTTCATACTAGGGTGCGACAGGTCGCTATCCCCTAGAATATCTACTCAA


OmpA_V1   361 TCCAGGGGTAAGGCGGAACCAGAGGTTCTGGTATATTCAAGCGACTTCAAAGAAGCGGAG
OmpA_V2   361 TCCAGGGGTAAGGCGGAACCAGAGGTTCTGGTATATTCAAGCGACTTCAAAGAAGCGGAG
OmpA_GV3  361 TCTAGGGGTAAGGCGGAACCAGAGGTTCTGGTATATTCAAGCGACTTCAAAGAAGCGGAG
OmpA_V3   361 TCCAGGGGTAAGGCGGAACCAGAGGTTCTGGTATATTCAAGCGACTTCAAAGAAGCGGAG
OmpA_GV1  361 TCTAGGGGTAAGGCGGAACCAGAGGTTCTGGTATATTCAAGCGACTTCAAAGAAGCGGAG
OmpA_GV2  361 TCTAGGGGTAAGGCGGAACCAGAGGTTCTGGTATATTCAAGCGACTTCAAAGAAGCGGAG


OmpA_V1   421 AAGGCGCATGCCCAAAACCGAAGGGTTGTACTAATCGTGGAGTGTCAGCACTCGGTATCT
OmpA_V2   421 AAGGCGCATGCCCAAAACCGAAGGGTTGTACTAATCGTGGAGTGTCAGCACTCGGTATCT
OmpA_GV3  421 AAGGCGCATGCCCAAAACCGAAGGGTTGTACTAATCGTGGAGTGTCAGCACTCGGTATCT
OmpA_V3   421 AAGGCGCATGCCCAAAACCGAAGGGTTGTACTAATCGTGGAGTGTCAGCACTCGGTATCT
OmpA_GV1  421 AAGGCGCATGCCCAAAACCGAAGGGTTGTACTAATCGTGGAGTGTCAGCACTCGGTATCT
OmpA_GV2  421 AAGGCGCATGCCCAAAACCGAAGGGTTGTACTAATCGTGGAGTGTCAGCACTCGGTATCT


OmpA_V1   481 CCCAAGAAAAAAATGGCTATCAAGTGGCCGTTCAGCTTTGGTAGGAGCGCGGCTAAGCAA
OmpA_V2   481 CCCAAGAAAAAAATGGCTATCAAGTGGCCGTTCAGCTTTGGTAGGAGCGCAGCTAAGCAA
OmpA_GV3  481 CCCAAGAAAAAAATGGCTATCAAGTGGCCGTTCAGCTTTGGTAGGAGCGCGGCTAAGCAA
OmpA_V3   481 CCCAAGAAAAAAATGGCTATCAAGTGGCCGTTCAGCTTTGGTAGGAGCGCGGCTAAGCAA
OmpA_GV1  481 CCCAAGAAAAAAATGGCTATCAAGTGGCCGTTCAGCTTTGGTAGGAGCGCGGCTAAGCAA
OmpA_GV2  481 CCCAAGAAAAAAATGGCTATCAAGTGGCCGTTCAGCTTTGGTAGGAGCGCGGCTAAGCAA


OmpA_V1   541 GATGATGTCGGTAGCAGCGAAGTTTCTGACGAAAATCCCGTCGACGATTCCTCAGAGGGG
OmpA_V2   541 GATGATGTCGGTAGCAGCGAAGTTTCTGACGAAAATCCCGTCGACGATTCCTCAGAGGGG
OmpA_GV3  541 GATGATGTCGGTAGCGGCGAAGTTTCTGACGAAAATCCCGTCGACGATTCCTCAGAGGGG
OmpA_V3   541 GATGATGTCGGTAGCAGCGAAGTTTCTGACGAAAATCCCGTCGACGATTCCTCAGAGGGG
OmpA_GV1  541 GATGATGTCGGTAGCAGCGAAGTTTCTGACGAAAATCCCGTCGACGATTCCTCAGAGGGG
OmpA_GV2  541 GATGATGTCGGTAGCAGCGAAGTTTCTGACGAAAATCCCGTCGACGATTCCTCAGAGGGG


OmpA_V1   601 ATAGCATCCGAAGAAGCGGCGCCTGAAGAAGGGGTGGTGTCCGAGGAAGCGGCGGAGGAG
OmpA_V2   601 ATAGCATCCGAAGAAGCGGCGCCTGAAGAAGGGGTGGTGTCCGAGGAAGCGGCGGAGGAG
OmpA_GV3  601 ATAGCATCCGAAGAAGCGGCGCCTGAAGAAGGGGTGGTGTCCGAGGAAGCGGCGGAGGAG
OmpA_V3   601 ATAGCATCCGAAGAAGCGGCGCCTGAAGAAGGGGTGGTGTCCGAGGAAGCGGCGGAGGAG
OmpA_GV1  601 ATAGCATCCGAAGAAGCGGCGCCTGAAGAAGGGGTGGTGTCCGAGGAAGCGGCGGAGGAG
OmpA_GV2  601 ATAGCATCCGAAGAAGCGGCGCCTGAAGAAGGGGTGGTGTCCGAGGAAGCGGCGGAGGAG


OmpA_V1   661 GCGCCGGAAGTGGCACAGGATTCTTCGGCAGGAGTGGTCGCGCCTGAATAG
OmpA_V2   661 GCGCCGGAAGTGGCACAGGATTCTTCGGCAGGAGTGGTCGCGCCTGAATAG
OmpA_GV3  661 GCACCGGAAGTGGCACAGGATTCTCCGGCAGGAGTGGTCGCGCCTGAATAG
OmpA_V3   661 GCACCGGAAGTGGCACAGGATTCTCCGGCAGGAGTGGTCGCGCCTGAATAG
OmpA_GV1  661 GCACCGGAAGTGGCACAGGATTCTCCGGCAGGAGTGGTCGCGCCTGAATAG
OmpA_GV2  661 GCACCGGAAGTGGCACAGGATTCTCCGGCAGGAGTGGTCGCGCCTAAATAG


S1 Fig. DNA alignment of all OmpA variants. Ghanaian OmpA variants include GV1, GV2 and GV3. OmpA_V1 represents St. Maries, Virginia, Kansas 6DE, Colville C51 and C52, and Nayarit-Mexico N3574. OmpA_V2 represents Puerto Rico, Dawn-Australia, and Nayarit-Mexico N4506. OmpA_V3 represents Kansas EMÖ.
