# Supplementary material for: Sequence and immunologic conservation of Anaplasma marginale OmpA within strains from Ghana as compared to the predominant OmpA variant
Source: PLoS One. 2019 Jul 10;14(7):e0217661. doi: 10.1371/journal.pone.0217661 (PMC6619652; doi:10.1371/journal.pone.0217661)
Supplement: S2 Fig — Ghanaian OmpA variants include GV1, GV2 and GV3. OmpA_V1 represents St. Maries, Virginia, Kansas 6DE, Colville C51 and C52, and Nayarit-Mexico N3574. OmpA_V2 represents Puerto Rico, Dawn-Australia, and Nayarit-Mexico N4506. OmpA_V3 represents Kansas EMΦ. (RTF) [file pone.0217661.s002.rtf]

OmpA_GV3    1 MLHRWLALCFLASFAVTGCGLFSKEKVGMDIVGVPFSAGRVEKVYFDFNKYEIKGSGKKV
OmpA_GV2    1 MLHRWLALCFLASFAVTGCGLFSKEKVGMDIVGVPFSAGRVEKVYFDFNKYEIKGSGKKV
OmpA_V1     1 MLHRWLALCFLASFAVTGCGLFSKEKVGMDIVGVPFSAGRVEKVYFDFNKYEIKGSGKKV
OmpA_V2     1 MLHRWLALCFLASFAVTGCGLFSKEKVGMDIVGVPFSAGRVEKVYFDFNKYEIKGSGKKV
OmpA_V3     1 MLHRWLALCFLASFAVTGCGLFSKEKVGMDIVGVPFSAGRVEKVYFDFNKYEIKGSGKKV
OmpA_GV1    1 MLHRWLALCFLASFAVTGCGLFSKEKVGMDIVGVPFSAGRVEKVYFDFNKYEIKGSGKKV


OmpA_GV3   61 LLGLVERMKADKRSTLLIIGHTDSRGTEEYNLALGERRANAVKEFILGCDRLLSPRISTQ
OmpA_GV2   61 LLGLVERMKADKRSTLLIIGHTDSRGTEEYNLALGERRANAVKEFILGCDRSLSPRISTQ
OmpA_V1    61 LLGLVERMKADKRSTLLIIGHTDSRGTEEYNLALGERRANAVKEFILGCDRSLSPRISTQ
OmpA_V2    61 LLGLVERMKADKRSTLLIIGHTDSRGTEEYNLALGERRANAVKEFILGCDRSLSPRISTQ
OmpA_V3    61 LLGLVERMKADKRSTLLIIGHTDSRGTEEYNLALGERRANAVKEFILGCDRSLSPRISTQ
OmpA_GV1   61 LLGLVERMKADKRSTLLIIGHTDSRGTEEYNLALGERRANAVKEFILGCDRSLSPRISTQ


OmpA_GV3  121 SRGKAEPEVLVYSSDFKEAEKAHAQNRRVVLIVECQHSVSPKKKMAIKWPFSFGRSAAKQ
OmpA_GV2  121 SRGKAEPEVLVYSSDFKEAEKAHAQNRRVVLIVECQHSVSPKKKMAIKWPFSFGRSAAKQ
OmpA_V1   121 SRGKAEPEVLVYSSDFKEAEKAHAQNRRVVLIVECQHSVSPKKKMAIKWPFSFGRSAAKQ
OmpA_V2   121 SRGKAEPEVLVYSSDFKEAEKAHAQNRRVVLIVECQHSVSPKKKMAIKWPFSFGRSAAKQ
OmpA_V3   121 SRGKAEPEVLVYSSDFKEAEKAHAQNRRVVLIVECQHSVSPKKKMAIKWPFSFGRSAAKQ
OmpA_GV1  121 SRGKAEPEVLVYSSDFKEAEKAHAQNRRVVLIVECQHSVSPKKKMAIKWPFSFGRSAAKQ


OmpA_GV3  181 DDVGSGEVSDENPVDDSSEGIASEEAAPEEGVVSEEAAEEAPEVAQDSPAGVVAPE*
OmpA_GV2  181 DDVGSSEVSDENPVDDSSEGIASEEAAPEEGVVSEEAAEEAPEVAQDSPAGVVAPK*
OmpA_V1   181 DDVGSSEVSDENPVDDSSEGIASEEAAPEEGVVSEEAAEEAPEVAQDSSAGVVAPE*
OmpA_V2   181 DDVGSSEVSDENPVDDSSEGIASEEAAPEEGVVSEEAAEEAPEVAQDSSAGVVAPE*
OmpA_V3   181 DDVGSSEVSDENPVDDSSEGIASEEAAPEEGVVSEEAAEEAPEVAQDSPAGVVAPE*
OmpA_GV1  181 DDVGSSEVSDENPVDDSSEGIASEEAAPEEGVVSEEAAEEAPEVAQDSPAGVVAPE*


S2 Fig. Amino acid alignment of all OmpA variants. Ghanaian OmpA variants include GV1, GV2 and GV3. OmpA_V1 represents St. Maries, Virginia, Kansas 6DE, Colville C51 and C52, and Nayarit-Mexico N3574. OmpA_V2 represents Puerto Rico, Dawn-Australia, and Nayarit-Mexico N4506. OmpA_V3 represents Kansas EMÖ.
